# Nano CrGeTe_3_: Topological Hall Effect in the Metallic State under High Pressure

**DOI:** 10.34133/research.0914

**Published:** 2025-10-23

**Authors:** Min Wang, Jijun Xue, Bo Zhang, Xiaoying Wang, Rusen Yang, Hua Pang

**Affiliations:** ^1^ School of Physical Science and Technology, Lanzhou University, Lanzhou 730000, China.; ^2^ State Key Laboratory of Low-Dimensional Quantum Physics and Department of Physics, Tsinghua University, Beijing 100084, China.; ^3^ School of Advanced Materials and Nanotechnology, Xidian University, Xi’an 710126, China.

## Abstract

The ferromagnetic (FM) properties of the van der Waals charge transfer insulator CrGeTe_3_ provide a promising avenue for concurrently controlling magnetic and electrical characteristics. This enables the potential integration of devices using nanoscale materials. In this research, we have effectively produced highly stable CrGeTe_3_ nanosheets by utilizing a Cr_2_Te_3_ template. Additionally, we show that the magnetic and electrical characteristics of CrGeTe_3_ single crystals can be continuously adjusted through the application of pressure. Electrical transport measurements reveal that CrGeTe_3_ transitions from an insulator under ambient conditions to a metallic state at 6.02 GPa. After reaching 25.33 GPa, CrGeTe_3_ undergoes a phase transition from the FM state to the Néel antiferromagnetic state at 30 K. This transition highlights the important impact of pressure on exchange interactions, corroborating density functional theory calculations. Micromagnetic simulation shows that the high-pressure topological Hall effect above 25.33 GPa originates from the Néel-type skyrmion. The interaction observed between magnetic couplings and high pressure provides crucial insights into the novel topological transport phenomena and their underlying physics, which is valuable for advancing spintronics devices and other emerging electronic technologies.

## Introduction

Two-dimensional van der Waals materials, which feature layered structures, have attracted considerable interest because of their promise as a flexible platform for exploring various physical phenomena by adjusting the film thickness [1–5]. Nevertheless, the lack of inherent ferromagnetism in traditional 2-dimensional (2D) materials has obstructed their development and usage in spintronic devices [6–11]. Inducing extrinsic ferromagnetism through strain engineering, edge modification, or defect introduction in 2D materials often presents marked control challenges [12–15]. There is a critical need to explore new 2D materials that possess inherent long-range ferromagnetism. CrGeTe_3_ has gained marked attention as a highly promising option because of its distinctive blend of semiconducting and ferromagnetic (FM) characteristics [16–20]. Layered transition-metal trichalcogenides (TMTC) with the chemical formula ABX_3_ have attracted recent interest as potential candidates for 2D magnets. In this regard, TMTC such as CrGeTe_3_ represents a rather attractive material [16,21,22]. The bilayer CrGeTe_3_ FM order was initially discovered by Gong and their team in 2017 [20]. Their study demonstrated that ferromagnetism persists even when the material is reduced to a thickness of several atomic layers or less, allowing for the realization of long-range FM interactions in CrGeTe_3_ nanosheets. The observation of ferromagnetism in CrGeTe_3_ heralds important opportunities for 2D materials in magnetic, magnetoelectric, and magneto-optic applications [23–28].

The energy gap in a Mott insulator can be adjusted through 2 primary methods: introducing charge carriers to fill the band or modifying the bandwidth, which induces an insulator-to-metal transition (IMT). This transition is frequently associated with structural and magnetic transformations. To date, doping has been achieved using approaches such as gating in field-effect transistor structures or inserting organic ions into bulk single crystals [24,29–31]. Research on few-layer CrGeTe_3_ [24] and CrI_3_ [29] utilizing electrostatic gating has shown the ability to adjust the coercive field and saturation magnetization. Nevertheless, when a high carrier concentration is introduced into CrGeTe_3_ through intercalation [31] or ionic liquid gating in FET structures [30], a $T_{c}$ (Curie temperature) of roughly 200 K is attained, accompanied by the stabilization of a metallic phase. Despite the encouraging nature of these findings, such doping-controlled techniques induce unwanted effects, such as substantial charge nonuniformity at the atomic scale. This can be attributed to either the disorder generated by intercalated ions or the chemical alterations caused by ionic liquid gating [32].

Pressure, as a conventional thermodynamic parameter, serves as an effective and noninvasive tool to modulate atomic and molecular distances, thereby influencing physical properties. In a range of materials, pressure serves to adjust the bandwidth and also alters spin exchange pathways by accurately modifying the distances and angles between atoms. This process effectively circumvents issues that stem from disorder. The application of pressure has successfully modulated the interlayer magnetism in few-layer CrI_3_ [33,34], as well as induced changes in $T_{c}$ for both CrI_3_ and CrGeTe_3_ bulk single crystals [35–37]. However, previous studies have primarily focused on pressures below 2.0 GPa, where the critical temperature ($T_{c}$) varies by approximately 10%. Notably, a recent high-pressure investigation of CrSiTe_3_ uncovered a simultaneous structural transition and IMT, followed by superconductivity at around 7.0 GPa [38]. Although the pressure-dependent lattice dynamics of CrGeTe_3_ have been studied, showing a transformation from phase I (space group: $R\bar{3}$; *a* = 6.4839 Å, *b* = 6.4839 Å, *c* = 17.670 Å; α = 90°, *β* = 90°, *γ* = 120°) to phase II (space group: R3; *a* = 3.9091 Å, *b* = 3.9091 Å, *c* =23.260 Å; *α* = 90°, *β* = 90°, *γ* =120°) around 18 GPa [35,39], the magnetic and electrical characteristics under high-pressure conditions are still largely unknown.

In this research, we managed to fabricate CrGeTe_3_ nanosheets using a chemical cation exchange approach. We characterized the FM properties of the CrGeTe_3_ nanosheets and systematically evaluated their electrical transport properties, including temperature-dependent resistance and the Hall effect, in single-crystalline CrGeTe_3_ under extreme pressures up to 35.33 GPa using a custom-designed diamond anvil cell (DAC). Furthermore, density functional theory (DFT) computations were performed under pressure to examine the electronic structure and magnetic ordering.

## Results

**Fig. 1.**
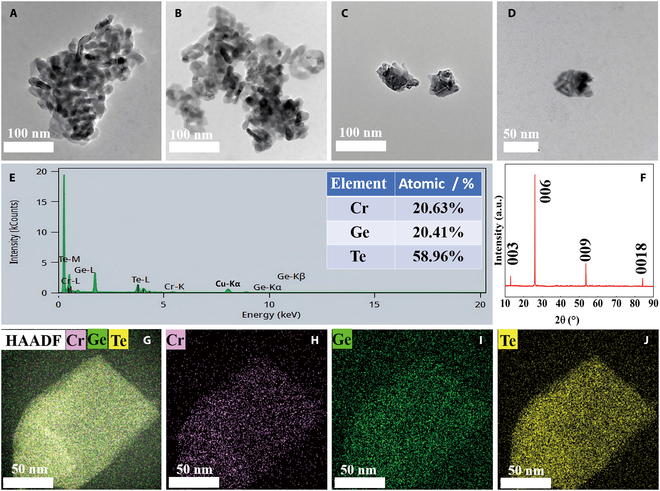
(A to D) TEM images of nano CrGeTe_3_ under ambient conditions; (E) EDS characterization; (F) XRD pattern of nano CrGeTe_3_; (G to J) elemental mapping images.

The morphologies of the materials were characterized using TEM as shown in Fig. 1A to D. The clavate-shaped CrGeTe_3_ materials display well-defined boundaries and smooth surfaces, indicative of a high degree of crystallinity in the initial sample. A closer examination reveals that the length of these microstructures is consistently below 100 nm. The irregular morphology of nanostructured CrGeTe_3_ observed under a transmission electron microscope is influenced by multiple factors, including the material’s intrinsic properties, sample preparation conditions, and external environmental conditions. Nevertheless, the sample remains within the nanoscale regime and does not compromise the accuracy of its classification as a nanoscale material. Additionally, EDS analysis (Fig. 1E) confirms an atomic ratio of Cr:Ge:Te to be approximately 1:1:3 for nano CrGeTe_3_. The structural characteristics of CrGeTe_3_ were examined via x-ray diffraction (XRD) as shown in Fig. 1F. The samples exhibit only sharp (00n) peaks, with no discernible diffraction peaks from impurities, indicating a high degree of crystallinity in the pristine CrGeTe_3_ nanosheets, which is consistent with previous studies [40–43]. Our XRD results provide characterization of the crystal structure of CrGeTe_3_ solely under ambient pressure conditions, which imposes certain limitations. Due to the absence of high-pressure synchrotron radiation diffraction experiments, we are unable to directly determine the crystal structure of CrGeTe_3_ under high-pressure conditions. A review of existing literature indicates that CrGeTe_3_ undergoes a structural phase transition from phase I to phase II under high pressure [39,44,45]. The results (Fig. 1G to J) reveal a uniform distribution of Cr, Ge, and Te throughout the structure.

**Fig. 2.**
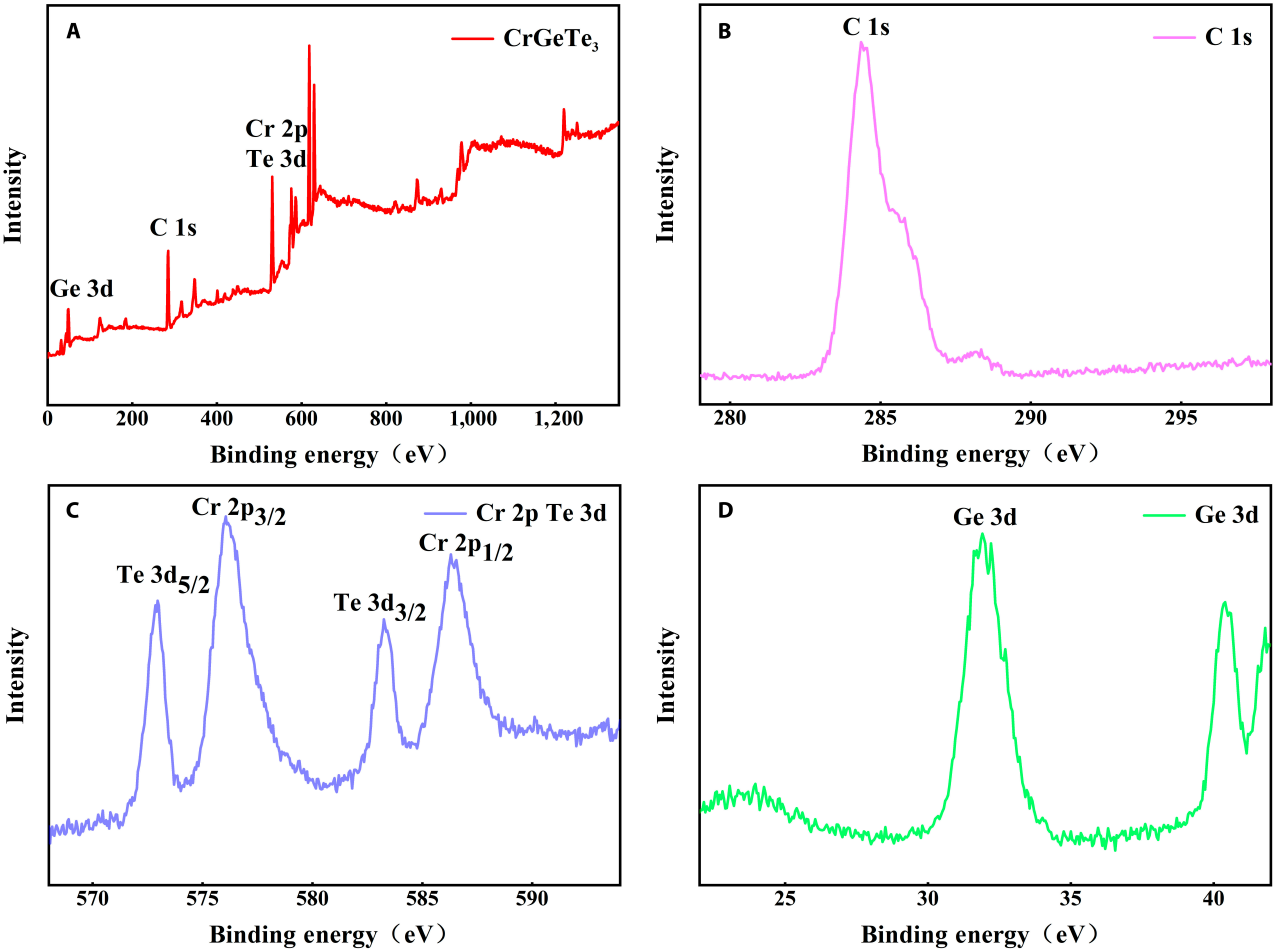
(A to D) XPS spectra of nano CrGeTe_3_: (A) survey spectrum; (B) C region; (C) Cr and Te region; (D) Ge region.

The detailed elemental composition of the prepared CrGeTe_3_ nanosheets is further characterized by x-ray photoelectron spectroscopy (XPS) measurements, with the results shown in Fig. 2A to D. The full XPS survey spectrum (Fig. 2A) indicates that the sample primarily consists of Cr (Cr 2p), Ge (Ge 3d), and Te (Te 3d). During XPS experiments, most samples exposed to the air can detect foreign carbon contamination. Upon contact with air, samples tend to adsorb certain carbon oxides or nitrogen oxides, which results in the detection of C, N, and O peaks even in samples that do not inherently contain these elements. The C peak (C–C peak) is calibrated at 284.8 eV to account for charge differences in Fig. 2B, followed by the calibration of other elements. Consequently, foreign carbon contamination is commonly employed as a reference for charge correction in XPS spectra. Figure 2C and D displays the detailed XPS spectra of Cr 2p, Ge 3d, and Te 3d. The peaks observed at 576.5 and 586.6 eV correspond to Cr 2p_3/2_ and Cr 2p_1/2_, respectively. Meanwhile, the satellite peaks at 577.5 and 587.6 eV correspond to Te 3d_3/2_ and Te 3d_5/2_, indicative of Cr^3+^ and Te^2−^ oxidation states, respectively [46]. For the Ge 3d spectrum (Fig. 2D), the peak observed at approximately 32.2 eV is consistent with the Ge^3+^ oxidation state [47].

**Fig. 3.**
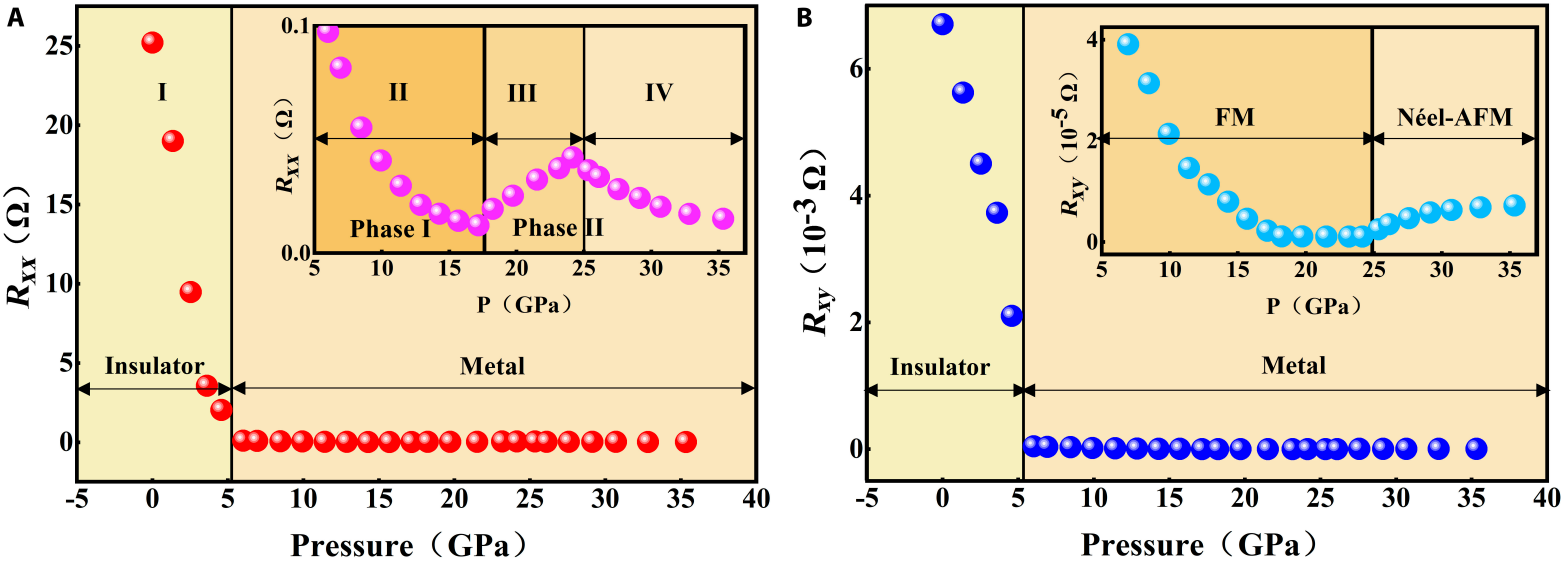
The dependence of longitudinal resistance *R_xx_*(A) and Hall resistance *R_xy_*(B) on various pressures is shown. The inset shows a magnification of the metal state.

The impact of pressure on the electronic characteristics and possible pressure-induced metallization of CrGeTe_3_ is explored further via electrical transport measurements. Due to the limited volume of the sample cavity, the measurement of sample thickness and diameter is prone to marked errors, which introduces distortion in the calculated resistivity data. Consequently, resistance is employed as an alternative metric to characterize changes in sample conductivity. We initially conducted resistance measurements of CrGeTe_3_ under pressure at 30 K, as illustrated in Fig. 3A. The *R*–*P* curves of CrGeTe_3_ can be categorized into 4 distinct stages: stage I (0 to 6.02 GPa), stage II (6.02 to 18.23 GPa), stage III (18.23 to 25.33 GPa), and stage IV (25.33 to 35.33 GPa). The resistance values at 0 GPa are approximately 25.2 Ω, indicating a highly insulating sample. Upon increasing pressure, the resistance decreases sharply from 25.2 Ω at 0 GPa to less than 0.0974 Ω at pressures exceeding 6.02 GPa. This marked reduction in resistance strongly suggests that CrGeTe_3_ undergoes a pressure-induced insulator–metal transition above approximately 6.02 GPa. This behavior is accurately reproduced by our subsequent DFT calculations. The resistance starts to gradually decrease with increasing pressure beyond 6.02 GPa. Previous angle-dispersive XRD experiments have clearly demonstrated the transformation of CrGeTe_3_ from phase I to phase II at a critical pressure of 18 GPa [35,39]. After 18 GPa, the dependence of resistance on pressure exhibits a marked change; resistance increases with increasing pressure up to 25.33 GPa, consistent with prior findings on the structural phase transitions of CrGeTe_3_ [35,39]. At 25.33 GPa, a notable inflection occurs, where resistance begins to decrease with further pressure increases up to 35.33 GPa, which is attributed to the transformation of magnetic states (for more details, see the section on magnetic properties calculations). Figure 3B present the results of Hall measurements conducted at 30 K and 0.2 T on nano CrGeTe_3_ under various applied pressures. The Hall resistance decreases markedly with increasing pressure up to 6.02 GPa. Beyond 6.02 GPa, the rate of decrease in Hall resistance markedly slows down. At 25.33 GPa, the Hall resistance at 30 K exhibits a monotonic increase with pressure, as illustrated in Fig. 3B. The measurement error in this experiment is 2.3%.

**Fig. 4.**
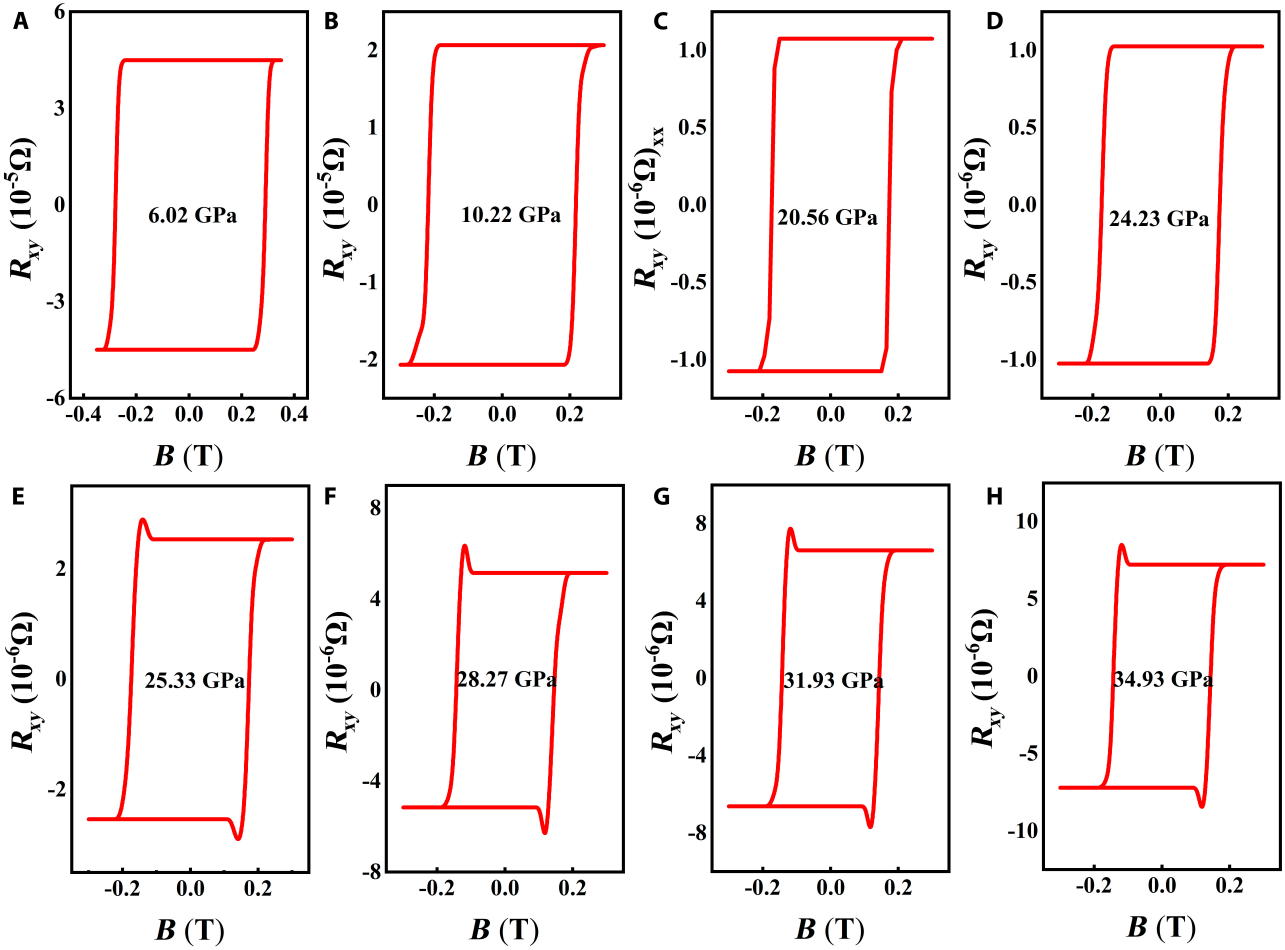
Magnetic-field dependence of $R_{\mathrm{xy}}$ at various pressures for the CrGeTe_3_ sample at 6.02 (A), 10.22 (B), 20.56 (C), 24.23 (D), 25.33 (E), 28.27 (F), 31.93 (G), and 34.93 GPa (H).

As shown in Fig. 4, the Hall resistance of CrGeTe_3_ exhibits dependencies on both pressure and magnetic field. Two notable features emerge from the data. First, the anomalous Hall signal undergoes a sign reversal across the entire tested pressure range. Second, pronounced hump-like structures appear during anomalous Hall effect (AHE) measurements. Specifically, additional bump structures are observed at magnetic fields corresponding to pressures of 25.33, 28.27, 31.93, and 34.93 GPa. Based on the literature reports [39], CrGeTe_3_ undergoes a structural transformation from phase I to phase II at 18.3 GPa. The diffraction peaks meanwhile became rather broadened as the pressure was further increased; the AM state started to appear at 26.5 GPa, indicating the concurrent pressure-induced amorphization. With the further increase of pressure up to 35.2 GPa, phase II disappears and the compound completely transforms into amorphous state. At 6.02 and 10.22 GPa, only phase I exists simultaneously in CrGeTe_3_. At 20.56 and 24.23 GPa, both phases I and II exist simultaneously in CrGeTe_3_. However, no hump structure is observed in the curves of Hall resistance varying with the magnetic field; that is, no topological Hall effect occurs. At 25.33 GPa, both phases I and II exist simultaneously in CrGeTe_3_. CrGeTe_3_ does not undergo amorphous transformation, but a hump structure still appears; that is, the topological Hall effect occurs. At 28.27, 31.93, and 34.93 GPa, both phase II and amorphous states exist simultaneously in CrGeTe_3_, and the topological Hall effect is still observed. Furthermore, based on a review of the literature, we identified that the topological Hall effect induced by skyrmions manifests as paired protrusions (humps) in the Hall resistance near the coercive field and is independent of the magnetic field direction. While amorphous transformation may result in a linear change in the Hall effect or an increase in background noise, it lacks a nonlinear response with distinct topological characteristics [48–50]. Therefore, we ruled out that the topological Hall effect of CrGeTe_3_ at 25.33 GPa was caused by structural nonuniformity. Such anomalies in the Hall effect have recently been reported in chiral magnets hosting a skyrmion phase and have been attributed to the topological Hall effect [51–54]. The measurement error in this experiment is 2.6%. These findings suggest the presence of noncollinear spin structures within a specific pressure range, potentially indicating magnetic skyrmions that contribute an unconventional component to Hall transport phenomena. It is worth noting that a comprehensive understanding of the mechanisms underlying hump-like structures necessitates further theoretical investigation. We employed DFT to investigate the response of CrGeTe_3_’s magnetic properties under external pressure and demonstrated that the Hall resistance can be attributed to the emergence of Néel-type skyrmions beyond 25.36 GPa. A detailed analysis of our calculations will be provided subsequently.

**Fig. 5.**
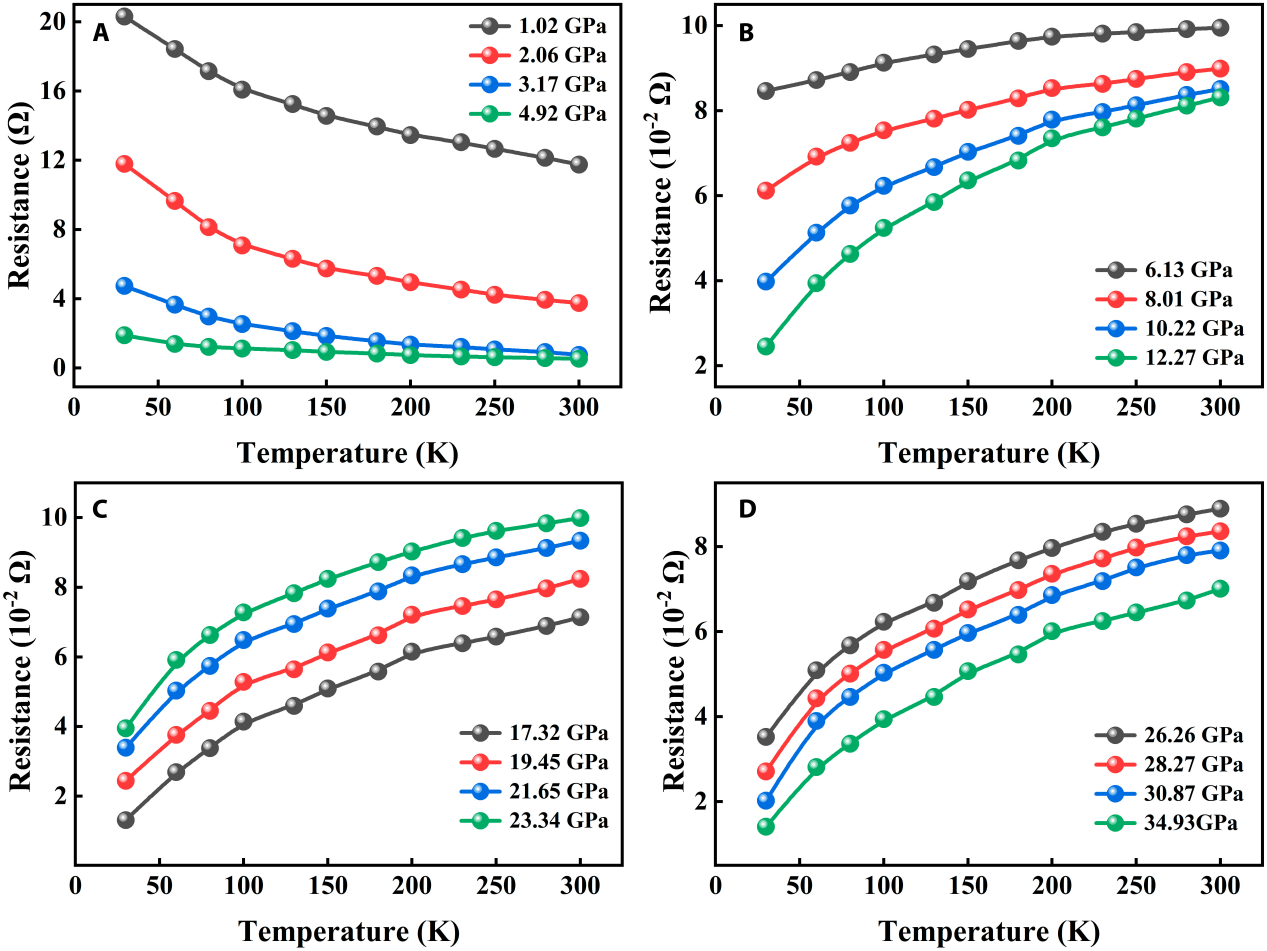
(A to D) Temperature dependence of resistance for CrGeTe_3_ at different pressures.

The connection between temperature and resistance serves as a reliable indicator of the material’s conductive properties. Consequently, we conducted measurements of temperature-dependent resistance under various pressures, as illustrated in Fig. 5. The data obtained from room temperature down to 30 K at pressures below 6.13 GPa show a decrease in resistance with increasing temperature, thereby confirming the insulating behavior of CrGeTe_3_ at low pressures. Additional compression leads to the suppression of the insulating characteristics. Temperature-dependent data gathered above 6.13 GPa show a positive slope, which verifies that CrGeTe_3_ enters a fully metallic state under high-pressure conditions. This metallization behavior is sustained throughout the measured pressure range. The measurement error in this experiment is 2.1%.

At normal pressure, the decrease in the resistivity index of CrGeTe_3_ is primarily driven by an increase in carrier concentration due to thermal activation. High pressure markedly modifies this behavior through the following mechanisms: suppressing the rise in carrier concentration and enhancing scattering effects, phonon softening and mode reconstruction intensifying electron–phonon scattering, and enhancement of magnetic order and spin-orbit coupling (SOC) altering transport characteristics. The synergistic interplay of these mechanisms results in the resistance of CrGeTe_3_ decreasing more gradually with increasing temperature under high pressure, exhibiting transport properties that are markedly distinct from those observed under normal pressure.

**Fig. 6.**
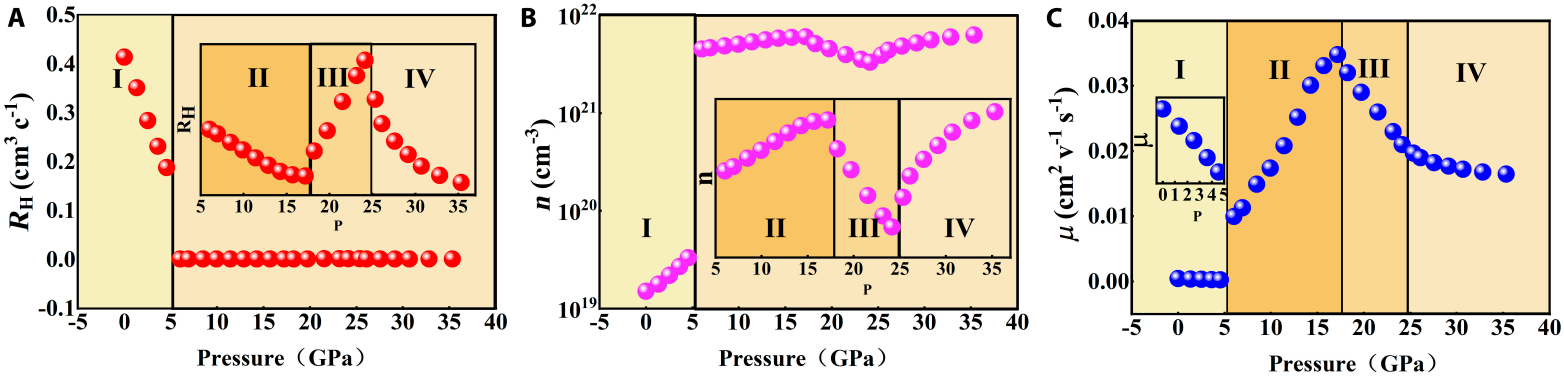
Pressure-dependent (A) Hall coefficient (*R*_H_), (B) charge carrier concentration (*n*), (C) and mobility (μ) of CrGeTe_3_ at 30K. The inset presents a magnification of the partial pressure stage.

To further investigate the charge carrier transport properties of CrGeTe_3_ under compression, the pressure dependencies of Hall coefficient ($R_{H}$), carrier concentration (*n*), and mobility (μ) were examined through in situ Hall effect measurements, as illustrated in Fig. 6. The underlying mechanisms governing the electrical transport properties under compression were analyzed based on these Hall effect measurements. The resistance at 30 K was determined based on the carrier concentration and mobility. The primary factors influencing carrier concentration are related to the effective mass at the Fermi surface, while phonon scattering from lattice vibrations predominantly affects mobility. An increase in carrier concentration reduces resistance, whereas a decrease in mobility enhances resistance. As illustrated in Fig. 6A, the Hall coefficient ($R_{H}$) remains positive over the entire pressure range. As illustrated in Fig. 6B and C, the carrier concentration increased by 2 orders of magnitude when the pressure reached 6.02 GPa, while the mobility exhibited a relatively gradual decrease. Consequently, in stage I (0 GPa to 6.02 GPa), the observed reduction in resistance with increasing pressure can be primarily attributed to the marked increase in carrier concentration. In stage II (6.02 GPa to 18.23 GPa), both the carrier concentration and mobility showed a gradual increase with rising pressure, leading to a further decrease in resistance. In stage III (18.23 to 25.33 GPa), the variations in carrier concentration and mobility with pressure exhibit opposite trends compared to those in phase II, albeit at distinct rates. The differing lattice structures result in varied free carrier scattering effects. The observed increase in resistance with increasing pressure during stage III can be attributed to reduced mobility, suggesting that the free carrier scattering effect was intensified following the phase transition. Beyond 25.33 GPa (stage IV), the electrical resistance decreases with increasing pressure, coinciding with a noticeable rise in carrier concentration. Meanwhile, the mobility of carriers exhibits a gradual decline. The observed decrease in resistance is primarily attributed to the increase in carrier concentration (*n*), which is partially counteracted by the concurrent decrease in carrier mobility (μ). The measurement error in this experiment is 2.3%. According to DFT, the abrupt changes in carrier concentration and mobility at 25.33 GPa may be indicative of a magnetic transition. In Fig. 6, it is observed that the sudden variations in the Hall coefficient, Hall mobility, and carrier concentration are strongly associated with the initiation of pressure-induced metallization, structural phase transitions, and magnetic transformations. Electrical measurements can effectively detect the nuanced changes in electrical parameters during phase transitions, demonstrating its feasibility. Nonetheless, relying solely on this approach is insufficient for exploring the possible relationship between phase structure and charge carrier transport. In order to fully comprehend the fundamental mechanisms of structural transitions and explain the unusual changes in electrical transport properties, the use of first-principles calculations is essential.

We conducted repeated measurements of longitudinal resistance, Hall resistance, Hall coefficient, carrier concentration, mobility at different pressures in the high-pressure Hall effect, as well as longitudinal resistance at different temperatures. The experimental rules were consistent, ensuring the repeatability of the high-pressure experimental data. The data were all acquired using the Lake Shore 8400 Hall effect measurement system. This system is a high-precision instrument for characterizing the electrical properties of materials, but errors may occur in actual measurements. Systematic errors arise from fixed calibration deviations, persistent contact inconsistencies, parasitic parameters, and inaccuracies in sample dimensions. These errors can be effectively corrected through proper calibration and optimization of the experimental setup. In contrast, statistical errors stem from random noise, temperature fluctuations, stochastic contact variations, and environmental interference. These can be mitigated by averaging multiple measurements and enhancing the signal-to-noise ratio. During actual measurements, it is essential to minimize overall error and enhance measurement accuracy by rigorously calibrating the instrument, managing environmental disturbances, and standardizing operational procedures.

**Fig. 7.**
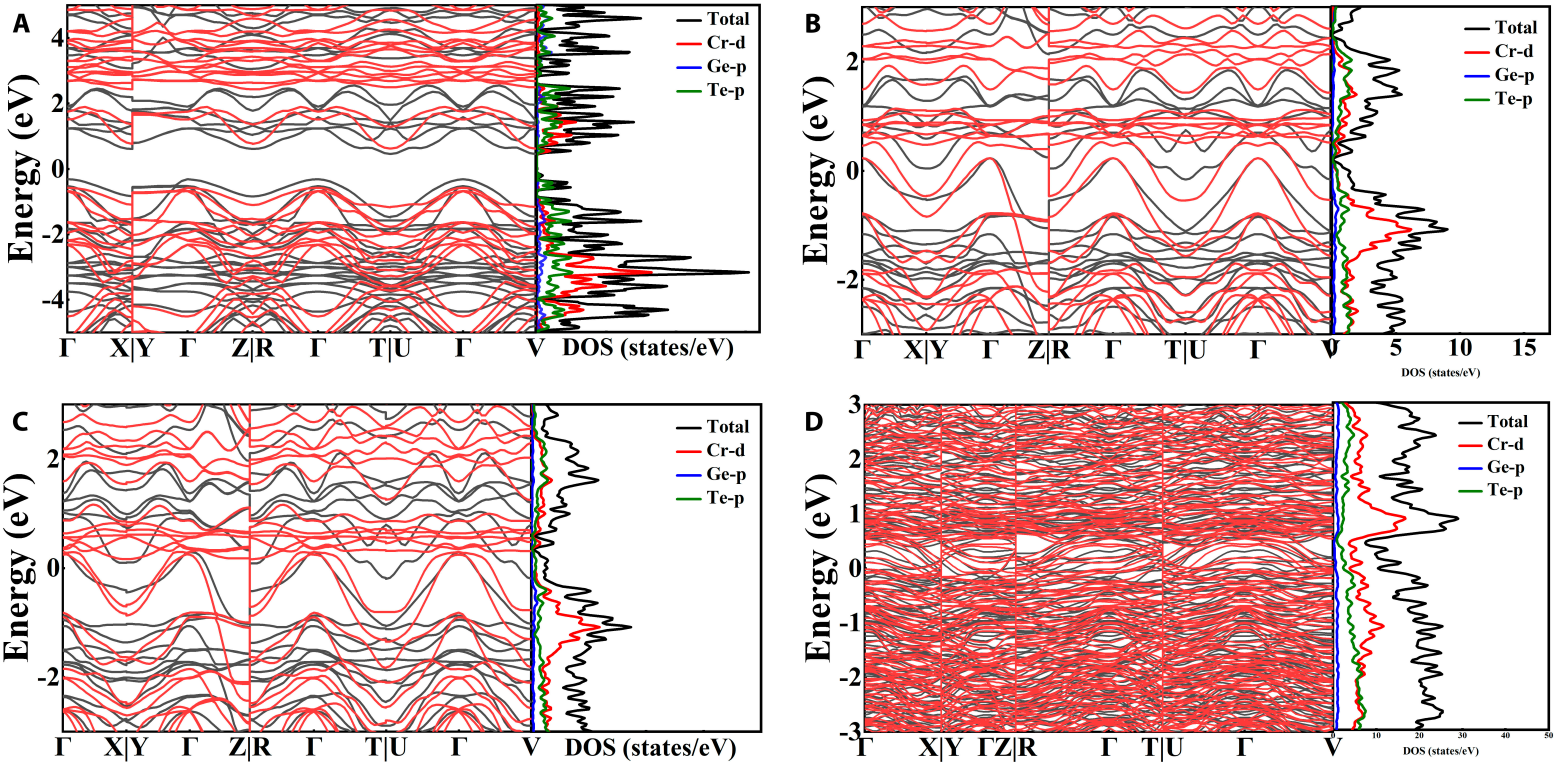
Band structures and DOS of CrGeTe_3_ calculated at various pressures: (A) 0 GPa, (B) 6 GPa, (C) 15 GPa, and (D) 25 GPa.

First-principles calculations can effectively investigate the potential correlation between phase structure and charge carrier transport, thereby aiding in the exploration of mechanisms underlying structural transformations and the root causes of anomalous changes in electrical transport properties. Figure 7 displays the band structure and partial density of states (DOS) under different pressures. At normal pressure, CrGeTe_3_ demonstrates a topological insulator phase. The highest point of the valence band is located at the Γ point, while the minimum of the conduction band is distributed around the T∣U points, suggesting an indirect band gap of 3.3 eV. As pressure rises, the band gap becomes narrower. As the IMT is approached at 6 GPa, the lower edge of the conduction band moves closer to the Fermi level, and the curvature at the bottom of the conduction band becomes more marked. These findings align with experimental results, which show that the resistance’s temperature dependence undergoes variations as pressure increases. The DOS indicates a finite electronic state distribution at the Fermi level (*E*_f_) in CrGeTe_3_ under 6 GPa, which is ascribed to the overlap between *E*_f_ and topologically trivial bands. The states above Ef are primarily associated with contributions from Cr-d and Te-p orbitals. As a result, the pressure-driven insulator-to-metal phase transition in CrGeTe_3_ is in excellent agreement with experimental findings. To gain further insight into the electronic structure under different pressures, Fig. 7C and D display the band structure and DOS at 15 and 25 GPa, respectively. According to the free-electron model, a larger DOS at the Fermi level [DOS(*E*_f_)] suggests a higher electron concentration. Therefore, the DOS(*E*_f_) can serve as an indicator of electron concentration in materials. Specifically, the DOS(*E*_f_) values are 12.84 eV at 25 GPa and 1.33 eV at 15 GPa. The concentration of electron carriers in CrGeTe_3_ peaks at 25 GPa, consistent with the findings from Hall measurements. Furthermore, the overlap between the conduction band and the valence band indicates that metallization is maintained at both 15 and 25 GPa.

In high-pressure experiments, the topological Hall effect is closely associated with the material’s crystal structure, electronic structure, and magnetic structure. High pressure induces changes in the crystal structure, such as reducing the lattice constant and decreasing atomic spacing. These structural modifications can influence the alignment of magnetic moments and magnetic interactions, thereby promoting the formation of a noncollinear magnetic arrangement, which provides favorable conditions for the emergence of the topological Hall effect. At the same time, the application of high pressure induces marked alterations in the electronic structure of the material. This could potentially lead to unique topological properties of the bands near the Fermi surface, such as the emergence of distinctive band structure features like Weyl points. Based on the aforementioned experimental analysis, it is evident that the crystal structure of CrGeTe_3_ transitions from phase I and phase II at 18 GPa. Additionally, the conduction band overlaps with the valence band at 6.02 GPa, thereby facilitating the insulator–conductor transition. However, no distinctive Weyl point band structure is observed at 25 GPa. The pressure values corresponding to crystal structure transitions and band structure transitions do not align with the pressure points associated with the topological Hall effect at 25 GPa. Consequently, we primarily attribute the transformation of the topological Hall effect at 25 GPa to the magnetic structure transition.

**Fig. 8.**
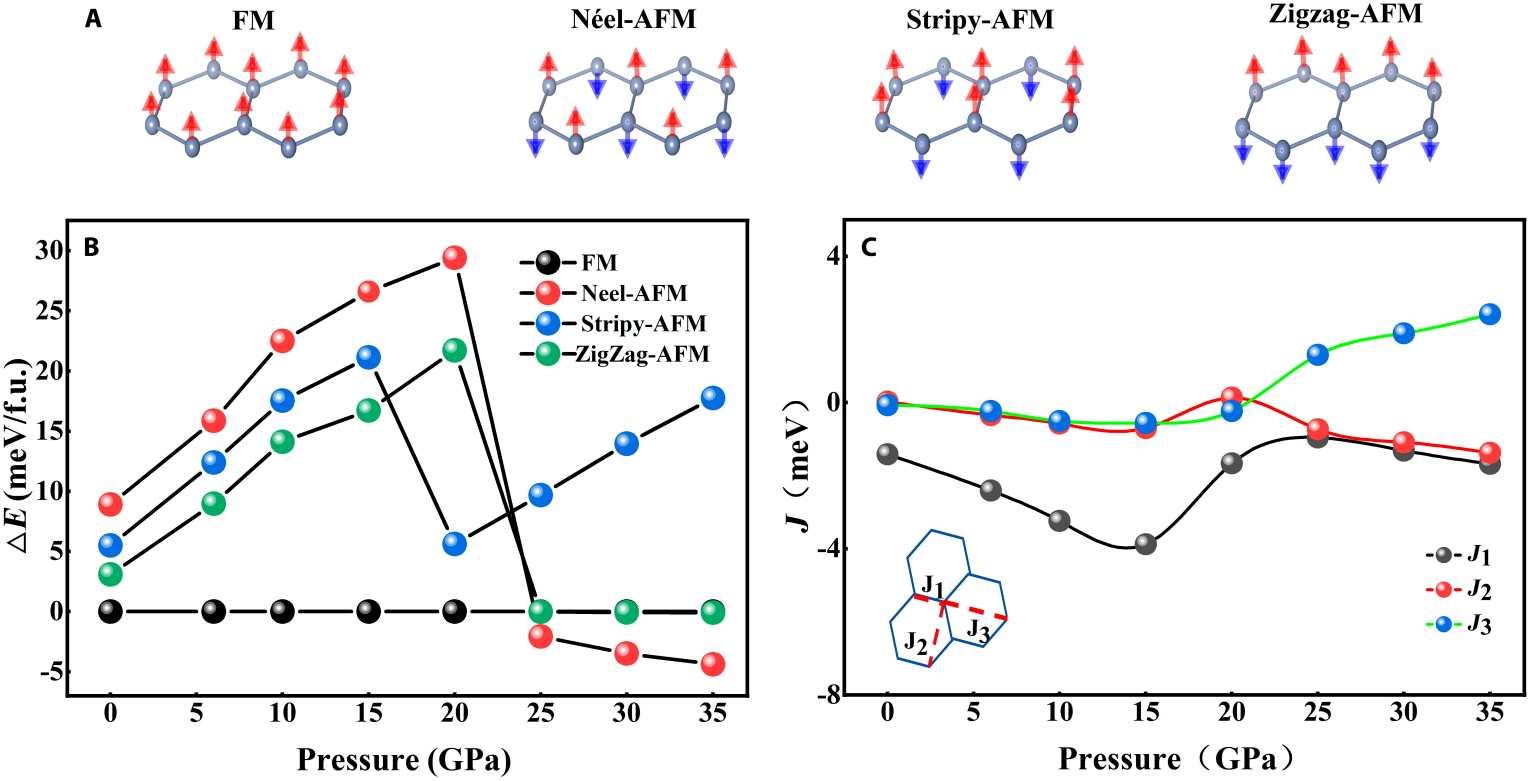
(A) Schematics of the ferromagnetic order and 3 types of antiferromagnetic orders: Néel-AFM, Stripy-AFM, and Zigzag-AFM. (B) The computed energy variations between different magnetic arrangements and the ground state ferromagnetic (FM) structure as pressure changes. (C) Estimated intralayer exchange couplings for the first, second, and third nearest neighbors ($J_{1}$, $J_{2}$, $J_{3}$), depicted in the insets, presented as functions of pressure.

To gain deeper insights into the experimental observations, DFT calculations were utilized to systematically investigate the magnetic responses of CrGeTe_3_ under external pressure. We examined multiple magnetic states, including the FM state and 3 intralayer antiferromagnetic (AFM) states with interlayer FM coupling. These 3 AFM states are designated as Néel-AFM (nAFM), Stripy-AFM, and Zigzag-AFM, respectively, based on their distinct intralayer arrangements of local magnetic moments, as illustrated in Fig. 8A. The energy values dependent on pressure for these states, computed using the DFT+U approach, are illustrated in Fig. 8B. This diagram evidently shows that the FM ordering continues to be the ground state at pressures lower than 25 GPa. Upon reaching 25 GPa, the magnetic structure transitions to nAFM, coinciding with the appearance of Néel-type skyrmions. These findings are consistent with experimental observations, which show the emergence of the topological Hall effect at 25 GPa.

The interactions between layers and within layers are essential in defining the magnetic ground state of CrGeTe_3_. To investigate this, we utilized Monte Carlo (MC) simulations grounded in the Heisenberg Hamiltonian $\begin{array}{c} H=\sum_{i<j}J_{\mathrm{ij}}S_{i}S_{j}+A\sum_{\mathrm{ij}}{\left(S_{i}^{Z}\right)}^{2}+ \end{array}$$\begin{array}{c} \sum_{i<j}B_{\mathrm{ij}}S_{i}^{z}\cdot S_{j}^{z} \end{array}$. Here, $S_{i}$ represents the spin operator of Cr at site i (which is 3/2 in our scenario). It is evident from the formula that the Hamiltonian *H* consists of 3 terms. The first term denotes the isotropic exchange interaction between 2 spins, *S_i_* and *S_j_*, where $J_{\mathrm{ij}}$ denotes the exchange coupling constant and is commonly referred to as the Heisenberg exchange. The second term represents the single-particle magnetic anisotropy effect. For a given material, the constant *A* is typically adopted that signifies the single-ion anisotropy constant, and the coefficient *A* does not carry a subscript. The third term signifies the anisotropic exchange interaction between the 2 spins, *S_i_* and *S_j_*, where $B_{\mathrm{ij}}$ denotes the anisotropic exchange constant, representing the Dzyaloshinskii–Moriya (DM) exchange interaction. In our analysis of exchange interactions, we took into account the intralayer exchange terms for the first, second, and third nearest neighbors, denoted as $J_{i}$ and $B_{i}$ (i= 1, 2, 3). However, the “*J_i_*” and “*B_i_*” terms, both single subscripts, differ in meaning from “*J_ij_*” and “*B_ij_*” in the formula. Specifically, the subscripts of *J_i_* (*i* = 1, 2, 3) indicate the order of nearest neighbors; for instance, the first nearest neighbor is denoted as *J*_1_, the second as *J*_2_, and the third as *J*_3_. Likewise, the anisotropic exchange interaction coefficient *B_i_* (*i* = 1, 2, 3) corresponds to the interaction between the *i*th nearest neighbors. These are depicted in the insets of Fig. 8C. The variation of $J_{i}$ with pressure is shown in Fig. 8C. Prior to reaching 25 GPa, the absolute values of $J_{1}$ markedly exceed those of $J_{2}$ and $J_{3}$. The contribution of $J_{1}$ in the Hamiltonian is more marked than that of $J_{3}$ at lower pressures. However, beyond 25 GPa, the strength of *J*_3_ surpasses that of $J_{1}$, resulting in a greater contribution from $J_{3}$ to the Hamiltonian. As a result, the intralayer first nearest-neighbor exchange interaction $J_{1}$ primarily controls FM behavior, while the intralayer third nearest-neighbor exchange interaction mainly induces nAFM. The increase in the intralayer third nearest-neighbor exchange interaction at high pressures has been associated with the detection of the topological Hall effect in experimental investigations.

To verify that the topological Hall effect originates from skyrmions, we performed micromagnetic simulations of the magnetic structure of CrGeTe_3_ under high pressure ranging from 25 to 35 GPa. The magnetic Cr^3+^ ions were arranged in a 2D honeycomb lattice using a 100 **×** 50 **×** 1 supercell configuration. The unit cell contains 2 inequivalent sites corresponding to 2 magnetic sublattices, *M*_1_ and *M*_2_, which form an AFM ground state. The lattice parameters were adopted from DFT theoretical calculations. Periodic boundary conditions were applied in the simulations, with a damping coefficient of 1 and a temperature of 0 K. The time step was set to 0.1 fs. All calculations started from a random initial state, and the configurations shown in the paper are those after 5 ns of relaxation. The Vampire program was employed to solve the LLG equation, and the Hamiltonian was defined as: $\begin{array}{c} H_{\mathrm{AFM}}=J\sum_{<i,j>}m_{i}\cdot m_{j}+ \end{array}$$\begin{array}{c} \begin{array}{c} \sum_{<i,j>}D\cdot \left(m_{i}\times m_{j}\right)-K\sum_{i}{\left(m_{i}^{z}\right)}^{2}+H_{z}\sum_{i}m_{i} \end{array} \end{array}$. Among them, *J* denotes the exchange interaction, ***D*** represents the DM interaction coefficient, *K* stands for the uniaxial magnetic anisotropy constant, *H*_z_ is the external magnetic field applied in the vertical direction, which is set to 0.2 T to mimic the experimental conditions, and ***m****_i_* is the unit vector describing the direction of the magnetic moment. Based on the results of first-principles calculations, the magnetic exchange interaction is pressure-sensitive, particularly *J*_3_. Above 30 GPa, *J*_3_ exhibits a sign opposite to that of *J*_1_, leading to magnetic frustration within the system. In the DFT-derived magnetic configuration, the energy difference between the Néel-type AFM state and the FM state is approximately −(*J*_1_ + *J*_3_). Therefore, for computational simplicity, only the effective nearest-neighbor interaction (*J*_eff_) is considered. The values at 30 and 35 GPa are 0.58 and 0.73 meV, respectively. Since the strong electron correlation effect is not accounted for in standard DFT calculations—and this effect typically enhances the exchange interaction between adjacent sites—the effective exchange coefficient is set to 1.0 meV.

Within the pressure range of 25 to 35 GPa, CrGeTe_3_ adopts the R3 space group structure and exhibits DM interaction due to the breaking of spatial inversion symmetry. For comparison, similar materials such as CrI_3_—also belonging to the R3 space group—have calculated DM coefficients of approximately *D* ≈ 0.1 to 0.5 meV. In CrGeTe_3_, the absence of heavy elements like iodine may weaken the SOC effect. However, lattice distortions could instead lead to a large electric potential gradient experienced by electrons, which is conducive to SOC. The estimated DM coefficient range is therefore 0.05 to 0.3 meV. The direction of the DM interaction follows Moriya’s rule. The theoretical value of the uniaxial magnetic anisotropy constant *K* for CrGeTe_3_ is 0.1 meV/atom; however, structural disorder or experimental preparation conditions may reduce this value, and thus, it is set to 0.03 to 0.05 meV/atom in the calculations.

**Fig. 9.**
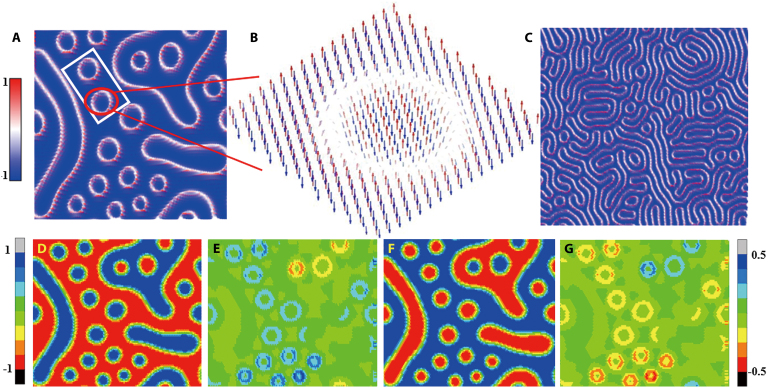
(A) Magnetic configuration at *J*_eff_ = 1.0 meV, *D* = 0.21 meV, and *K* = 0.05 meV. (B) Magnified view of the boxed region in panel (A). (C) Magnetic configuration at *J*_eff_ = 1.0 meV, *D* = 0.4 meV, and *K* = 0.05 meV. (D) The distribution of the *m*_z_ component and (E) the local chirality of magnetic sublattice *M*_1_; (F) and (G) are those for sublattice *M*_2_ of the honeycomb lattice. In the skyrmion regions, the signs of *m*_z_ components and local chiralities of the 2 sublattices are opposite, which is a characteristic of AFM Néel-type skyrmions. The local chirality is defined as $C_{i}=\sum_{\mathrm{jk}}S_{i}\cdot \left(S_{j}\times S_{k}\right)$, where *j* and *k* are the nearest neighbors of spin *i* and are also nearest neighbors to each other.

The calculation reveals that within the range of 0.17 < *D*/*J* < 0.67, the system can exhibit Néel-type skyrmions: when *D* is relatively small, isolated, circularly symmetric skyrmions are scattered; when *D* is larger, distorted skyrmions appear (Fig. 9A and B, *D* = 0.31). Upon further increasing *D*, worm-like domains also emerge in the magnetic structure (Fig. 9C, *D* = 0.37). Additionally, at the edge of the skyrmion (the white region) in Fig. 9B, the spin exhibits a radial distribution, a characteristic of Néel-type skyrmions. We have also provided additional global information about the magnetic configuration. The simulated system is a honeycomb lattice, with 2 inequivalent crystal sites in each unit cell. Under AFM coupling, these sites belong to 2 magnetic sublattices, *M*_1_ and *M*_2_. We present the distribution of the spin *z* component in each magnetic sublattice under the stable skyrmion state (Fig. 9D and F) and calculate the local chirality of each spin (Fig. 9E and G), defined as usual, $\;C_{i}=\sum_{\mathrm{jk}}S_{i}\cdot \left(S_{j}\times S_{k}\right)$ (where *j* and *k* are the nearest neighbors of spin *i* and are also nearest neighbors to each other). Figure 9D and F clearly show that the spatial distribution of the *z* component of the spins in the 2 magnetic sublattices fully aligns with AFM coupling, particularly in the skyrmion core region, where the distributions are just opposite, confirming the presence of 2 AFM-coupled skyrmions. The distribution of local chirality shows that, within the same skyrmion region, the chirality of the 2 magnetic sublattices is opposite. Considering the direct association between topological charge and chirality, each magnetic sublattice contributes a topological charge with a distinct sign. Therefore, the computational results indeed correspond to AFM Néel-type skyrmions. Our micromagnetic simulation provides evidence for the experimental observation of the high-pressure topological Hall effect above 25.33 GPa. In combination with the energy calculations of magnetic structures, the intra-layer nearest-neighbor exchange interaction, and the influence of the amorphous state on the Hall effect presented in the manuscript, it can be comprehensively concluded that the topological Hall effect observed experimentally originates from the Néel-type skyrmion (AFM) magnetic structure induced by high pressure.

## Conclusion

In summary, CrGeTe_3_ nanosheets were synthesized via a bottom-up chemical cation exchange method. The pressure-dependent FM properties of CrGeTe_3_ flakes were thoroughly examined using high-pressure Hall measurements and supported by theoretical calculations. Electrical transport studies indicate that CrGeTe_3_ transitions from an insulator under ambient conditions to a metallic state at 6.02 GPa. After reaching 25.33 GPa, CrGeTe_3_ undergoes a phase transition from FM to nAFM at 30 K. Our computations suggest that the pressure-induced magnetic behavior in CrGeTe_3_ is primarily determined by the intralayer third nearest-neighbor exchange interaction. Micromagnetic simulation provides that the high-pressure topological Hall effect above 25.33 GPa originates from the Néel-type skyrmion. These findings provide crucial insights into the mechanism of magnetic exchange interactions and pave a novel pathway for effectively modulating the magnetic properties of nano materials. These results offer essential perspectives on the mechanism of magnetic exchange interactions and open up a new approach for efficiently adjusting the magnetic characteristics of nano materials.

## Materials and Methods

The CrGeTe_3_ nanostructure was fabricated using a 2-step approach. Initially, a Cr_2_Te_3_ binary template was created by employing a modified organic-solvent-phase chemical decomposition technique, with a Cr:Te molar ratio set at 1:1.8. In the second step, the ternary CrGeTe_3_ compound was created by incorporating a Ge source into the pre-existing Cr_2_Te_3_ seed crystals via cation exchange. In particular, a suspension of 1 mmol Cr_2_Te_3_ seed crystals was introduced into a flask with 30 ml of oleic acid (OLA). Following this, the setup was purged with nitrogen gas at room temperature for half an hour to remove any remaining low-boiling-point solvents and oxygen. A transparent, colorless solution was prepared by dissolving 1 mmol of GeI_4_ in 5 ml of OLA. This solution was subsequently added dropwise to the mixture at 100 °C while stirring vigorously. Once the reflux temperature of 330 °C was attained at a heating rate of 5 °C per minute, the reaction mixture was kept at this temperature for an additional 2 h under continuous stirring. After the reaction, the solution was promptly cooled to ambient temperature, followed by an extraction and purification process analogous to that employed for Cr_2_Te_3_. The resulting CrGeTe_3_ nanostructures exhibited good dispersibility in hexane [55].

**Fig. 10.**
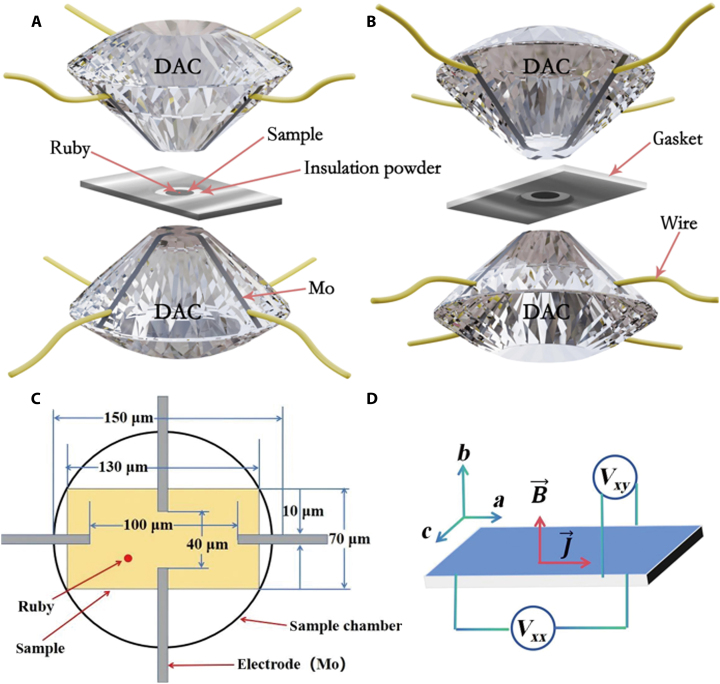
(A and B) Structure diagram of 4 electrodes, sample, insulation powder, and ruby in diamond anvil. (C) Top view of the sample chamber; (D) schematic drawing of the sample used in the experiments.

The experimental setup utilized a DAC to generate high pressures, featuring anvils with 300-μm-diameter facets [56–58]. A pre-indented T301 stainless steel gasket was used, and the sample chamber had dimensions of 150 μm in diameter and 50 μm in thickness. The configuration of electrodes, samples, insulation powder, and ruby within the DAC is depicted in Fig. 10A and B, shown from 2 distinct perspectives. Figure 10C shows a top view of the sample chamber. The configuration of a microcircuit on a diamond anvil used in our experiment comprises 4 molybdenum electrodes deposited on the surfaces of 2 diamond anvils via magnetron sputtering and photolithography. The sample, placed in the sample chamber, has dimensions of 130 μm in length and 70 μm in width. The Mo electrode features a width of 10 μm, with a longitudinal electrode distance of 100 μm and a transverse electrode distance of 40 μm. This setup is specifically designed to measure electrical transport properties under high-pressure conditions. The thin-film electrodes ensure that the position and shape of the sample’s electrodes remain stable during high-pressure experiments. When introducing the sample into the sample cavity, we typically employ a ruby as a pressure calibration material. Figure 10D provides a schematic illustration of the experimental sample. Longitudinal resistance ($R_{\mathrm{xx}}$) and Hall resistance ($R_{\mathrm{xy}}$) were measured using a 4-probe configuration, utilizing a Lake Shore 8400 system. Pressure calibration was conducted using a ruby chip, with pressure determination based on the observed shift in the ruby R1 fluorescence line [59–61]. The entire DAC assembly was then mounted onto the cold finger of the low temperature cryostat.

The phase of the synthesized samples was characterized by XRD technology. The x-ray diffractometer model was the German Bruker D8 Advance. The Cu Kα ray (wavelength, 1.5418 A) was selected. The scanning step was 0.02°, the scanning speed was 2°/min, the scanning angle range was 10°to90°, the working voltage was 40 kV, and the working current was 40 mA. In situ resistance and Hall effect measurements under high pressure were performed by employing the current reversal technique to remove thermoelectric offsets. A Keithley 2400 source meter supplied a current of approximately 100 nA, while a Keithley 2700 multimeter was employed for voltage measurement. The measurement procedure was carried out automatically by employing the van der Pauw technique. Furthermore, a magnetic field of 0.2 T was generated using an EM7 electromagnet, a 9060 electromagnet power supply, and a Lake Shore 8400 Gaussmeter. For the resistance measurements that depend on temperature, liquid helium was utilized to attain a temperature range of 4 to 300 K.

All DFT calculations were performed using Vienna Ab-initio Simulation Package (VASP) [62,63], adopting the generalized gradient approximation with the Perdew–Burke–Ernzerhof functional [64].The pressure was set via the PSTRESS parameter in the VASP software, and the 3-layer initial crystal structure of CrGeTe_3_ was selected for calculation. The $R\bar{3}$ phase was employed for pressures below 18 GPa, while the R3 phase was utilized for pressures above 18 GPa. The ionic cores were modeled using projected augmented wave (PAW) potentials [65,66], and the valence electrons were depicted with a plane wave basis set, employing a kinetic energy cutoff of 450 eV. Partial occupancies of the Kohn–Sham orbitals were allowed using the Gaussian smearing method with a smearing width of 0.02 eV. The electronic energy was considered self-consistent when the change in energy fell below 10^−5^ eV. The orthogonal supercell of CrGeTe_3_ was constructed based on the primitive cell. During structural optimizations, a 2 × 4 × 1 gamma-point centered k-point grid was employed for Brillouin zone sampling, and both atomic positions and lattice parameters were fully relaxed.

## Data Availability

The data used to support the findings of this study are available from the corresponding authors upon request.
