# Supplementary material for: Nano CrGeTe3: Topological Hall Effect in the Metallic State under High Pressure
Source: Research (Wash D C). 2025 Oct 23;8:0914. doi: 10.34133/research.0914 (PMC12547888; doi:10.34133/research.0914)
Supplement: Supplementary 1 — Figs. S1 and S2 [file research.0914.f1.docx]

**Supplementary Materials**


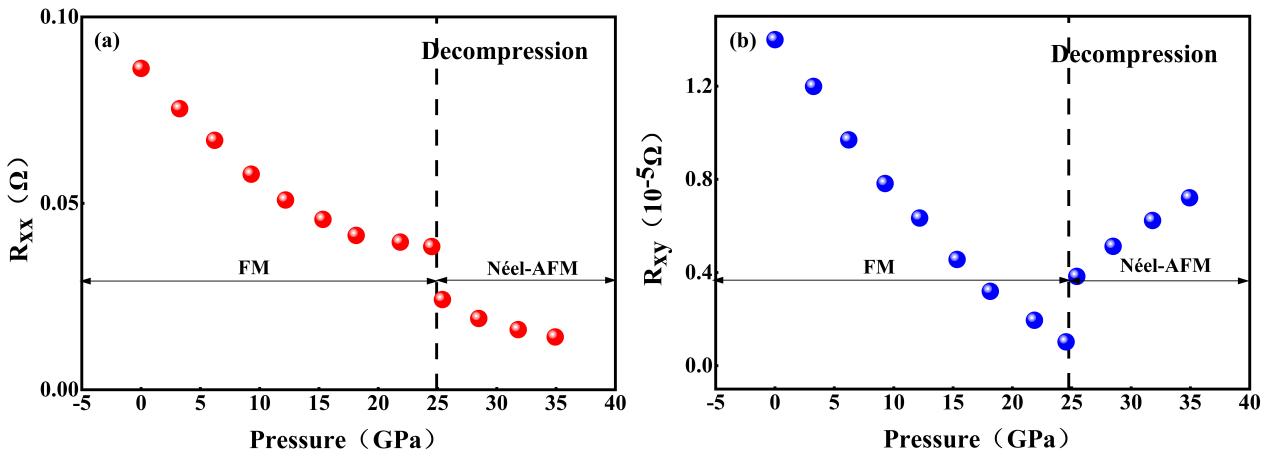


Figure S1. The variation relationship of longitudinal resistances R_xx_ and R_xy_ with pressure during the unloading process.

We supplemented the experimental data for the previous samples during the unloading process. Figure S1 illustrates the variation of longitudinal resistance R_xx_ and Hall resistance R_xy_ with pressure during the unloading phase of our experiment. As shown in the Figure S1, the longitudinal resistance R_xx_ continuously increases as the pressure decreases. Below 25.42 GPa, the resistance abruptly rises. After unloading to atmospheric pressure, the longitudinal resistance reaches 0.0862 Ω. Throughout the entire unloading process, the sample remains in a metallic state without undergoing a metal-insulator transition. The variation trend of the Hall resistance R_xy_ with pressure can be divided into two distinct stages. During the unloading from 34.93 GPa to 25.42 GPa, the Hall resistance gradually decreases with decreasing pressure. When the pressure drops below 24.52 GPa, the Hall resistance begins to increase gradually until reaching 0 GPa. However, compared to the initial unpressurized state, the magnitude differs by approximately two orders of magnitude. As shown in Figure S1, the variation trends of both longitudinal resistance R_xx_ and Hall resistance R_xy_ during the unloading process are markedly different from those observed during the pressurization process, and the experimental data do not reverse. Given that the electrical transport properties can partially reflect changes in crystal structure, and according to literature report^[41]^, CrGeTe_3_ remains amorphous after unloading, we propose that the observed variation patterns of R_xx_ and R_xy_ during unloading indicate that the sample retains its amorphous state throughout the process.


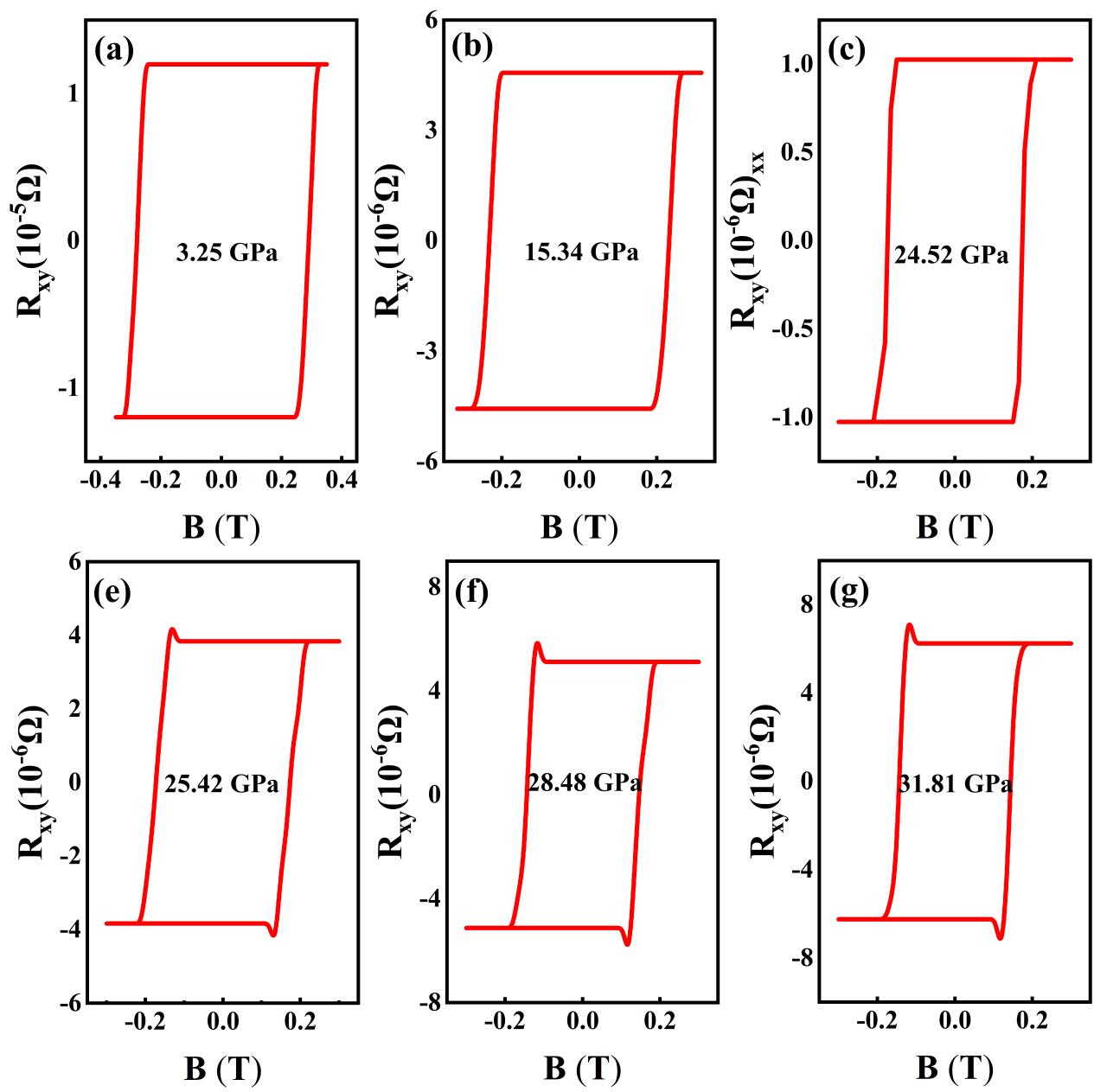


Figure S2. The variation relationship of Hall resistance R_xy_ at typical pressure points during the unloading process with the magnetic field.

The sample remained amorphous throughout the entire decompression process. However, in the Hall resistance R_xy_ versus magnetic field curves obtained during decompression (see Figure S2), two distinct features were observed. Prior to decompression down to 25.42 GPa, the curves exhibited a hump structure, which is indicative of the topological Hall effect. Below 24.52 GPa, the hump structure disappeared, suggesting the dominance of the anomalous Hall effect. These observations indicate that the amorphous state of CrGeTe_3_ is not responsible for the emergence of the topological Hall effect. The experimentally observed topological Hall effect should come from the topological nature of the magnetic structure.
